# The Institutional Worker

**Published:** 1919-10-18

**Authors:** 


THE
### INSTITUTIONAL WORKER
Being a Special Supplement to "The Hospital"

### OUR BUREAU OF INFORMATION.

### Rules for Correspondents.

1. Every letter must be accompanied by the coupon to be cut from the back cover (inside page) of The Hospital, current issue, and must contain the name and address of the correspondent with pseudonym for publication if desired. Replies by post cannot be given save under exceptional circumstances at the Editor's discretion.

2. Letters from Approved Homes in reply to special needs published in the Bureau should state terms and full particulars, and be sent prepaid under cover to the Editor of the Bureau with name written across coupon for identification.

3. Proprietors of Homes which have not yet been entered on the List of Approved Homes, but have spare accommodation likely to suit special needs, are invited to write for an application form for registration. The fee for registration, which includes two announcements of the Home in the Bureau and other privileges, is 10s.

4. All communications to be addressed to the Editor of The Hospital, 28 Southampton Street, Strand, London, W.C. 2, and marked "Bureau of Information."

### INSTITUTION FACTS AND FIGURES.

### Social Work for V.A.D.s.

#### HOSPITAL ALMONING.

By H. M. N.

Unlike many branches of social work, Hospital Almoning does not present an opening for either amateur or part-time helpers. It is highly trained work, and requires rather exceptional qualifications in those undertaking it. At the same time it is deeply interesting, and the almoner is yet to be met who does not love her work.

The object for which Lady Almoners are attached to the great hospitals, not only of London, but of an increasingly large number of provincial towns, is twofold. It is first to check the tremendous overcrowding of the out-patient department by weeding out those who ought not to be there. Such patients may be classed under three heads. First, those whose attendance is little short of a deliberate fraud, since they are quite well able to pay for medical advice in their own homes. This class is steadily decreasing, but there will always be people who will seize any opportunity for getting something for nothing. Secondly, those for whom other medical facilities exist, of which they should avail themselves before resorting to a hospital—insured persons, for instance, suffering from ordinary troubles with which their Panel doctors can deal. Thirdly, patients who are in such a destitute state that hospital treatment can do them little good, and for whom the Poor Law, with possible admission to workhouse or infirmary, is really the only solution.

But this weeding out is only a small part of the almoner's work, for when her enquiries and investigations have removed every unsuitable applicant, there will remain a huge body of accepted patients for whom her help will be required in a hundred different ways. It is obvious that when a patient goes to a hospital, and is seen by a busy doctor, who prescribes certain treatment, there is no certainty that that treatment will, or even can, be carried out unless there is some link between the hospital and the home behind the patient. That link is supplied by the almoner. She interviews the patient and then herself gets into touch with suitable agencies near the home: a health visitor or district nurse is requested to visit; a confidential report is obtained from the local Charity Organisation; a church or chapel in the district is requested to help with extra milk or other nourishment. In "At the Works" Lady Bell has pictured very clearly what serious or long-continued illness means to the poor, and the difficulty, almost impossibility, of their carrying out the medical advice received. Now, if a mother with "a bad leg" is told to rest it, an anæmic child is ordered into the country, or the breadwinner is advised to "come in" for an operation, it is the almoner who has to smoothe away the obstacles and make it possible for those desirable things to be done. Otherwise a great deal of the doctor's time and skill must inevitably be thrown away. It follows that the almoner must be a real friend to the patients, able to gain their confidence by her tact and sympathy. An in-patient may have "something on his mind" which disturbs him at night; the doctor may suspect that there is some complication in the domestic life of an out-patient which she is too shy to tell him. The almoner may be able to draw it forth and to make some practical suggestion.

But as well as the confidence of the patients, the almoner must be able to win the confidence of the nurses and doctors, and this is where a V.A.D. would probably find her war work specially helpful. She is accustomed to hospital etiquette and its rules and regulations, she knows the status of the various members of the staff and the confidential nature of diagnoses and medical opinions. She is also used to scientific terms, and the lectures in hygiene, etc., which form part of the training, should be of special interest to her. All information can be obtained from the Secretary of the Almoners' Council, Denison House, Vauxhall Bridge Road. The training takes eighteen months, and the fee is twenty-five guineas, but it should be remembered that this is one of the openings for which the Red Cross proposes to give scholarships to suitable members. Part of the training is given in the offices of the C.O.S., and part at a hospital under an almoner. There are also the lectures already referred to. It is necessary for the candidate to obtain experience by actual visiting in the homes of those from among whom her future patients will come: she also interviews and takes down particulars of cases at the office; she writes letters and keeps accounts, since a Samaritan Fund, of varying size, is available at most hospitals for use in the almoner's work. In addition, it is essential that she should be a veritable encyclopædia of charities and social agencies. She must know what openings for co-operation exist and to whom she will be able to refer her hospital cases; she must also know the benefits available under the Insurance Act and the Workmen's Compensation Act, etc., while it is only necessary to suggest what an enormous field is now opened by pensions.

Enough has been said to show that in almoning lies a work which will demand the best gifts of heart and brain that an educated woman can bring to it—will demand, but will also repay

Previous articles appeared July 19, Sept. 13 and Oct. 4.

### Question for October.

**REGULATING A PATIENT'S VISITORS.**

**Elaborate a plan whereby the number of patients' visitors may be regulated on visiting days, so that none may be standing about the corridors and all may see the patient in turn. Each patient to be allowed six visitors, but not more than two at a time.**

**(A minimum payment of 10s. 6d. will be made for each published answer.)**

### QUESTIONS INVITED.

IN connection with our Question Box, we offer every month five shillings each for the best questions which are sent in for consideration. The questions may be on any institutional subject and concern any department, from the secretary's to the porter's, or the matron's to the domestic staff; the one essential is that they must be practical and deal with points that have a definite relation to hospital or institutional administration, upkeep, finance, or management. Points relating to artificers' work, housekeeping, the laundry, out-patients, and other practical matters will be welcome. It is hoped that thus, when difficult or doubtful points arise in the course of their work, institutional workers will be encouraged to put them in the form of a question and send them to the Editor, THE HOSPITAL Bureau of Information, 28 & 29 Southampton Street, Strand, London, W.C. 2, marked "Question Box."

The best questions will be published in due course and our readers will be given the opportunity of answering them. Thus all institutional workers may be able to cooperate with the view of helping the work and smoothing the difficulties of each other. In short, we want the Question Box of the *Institutional Worker* to be freely used by every worker habitually.

**The Editor will be glad to receive correspondence and to consider contributions upon all subjects relating to institutional work which affect the welfare of institutional workers.**

### ENQUIRIES AND ANSWERS.

### *THE SICK AND IN NEED.*

### Home for Mentally Defective Girl in Liverpool.

PROBABLY one of the following homes will accept the case you refer to: The Adcote Home, Knotty Ash, Liverpool. This home is for feeble-minded girls of fifteen to seventeen years of age, who have sufficient brain-power to be trained. The payment is usually 9s. per week. Or the Horticultural School for Feeble-minded Girls, Dovecot, Knotty Ash, Liverpool. Inmates are trained in horticulture, house and laundry work; payment is from 10s. to 14s. weekly, and £3 for outfit. Apply to the Hon Secretary in each case.—SARAH.

### *EMPLOYMENT AND TRAINING.*

### Private Work for Mental Nurse.

THE address of the offices of St. Luke's Hospital is 19 Nottingham Place, London, W. There are many nursing associations in London which employ mental nurses, among them the Auxiliary Nurses' Society, 10 Orchard Street, W. A list of Associations is given in the book, "How to Succeed as a Trained Nurse," obtainable of The Scientific Press, Ltd., 28 and 29 Southampton Street, Strand, W.C. —L. M.

### Post as Children's Nurse.

WE can do no more than advise you to persevere in replying to advertisements which appear not only in the nursing journals, but in the daily press. You might also advertise your need.—M. C.

### Epilepsy.

A GREAT deal has been written upon this subject. If you will write to the Manager, The Scientific Press, Ltd., 28 and 29 Southampton Street, Strand, W.C. 2, he will send you a list of publications dealing with it.—MALE NURSE.

### League of Red Cross Societies.

THE League of Red Cross Societies will not in any way supersede the activities of the national societies—on the contrary its purpose is to systematise the efforts of the Red Cross Societies of the world to relieve suffering resulting from disease and calamity. It sets out to stimulate their peace-time activities, and to place at their disposal the latest knowledge and approved practices of the experts in public health and preventive medicine throughout the world. The League was inaugurated in Paris on May 5, 1919, under the chairmanship of Mr. Henry P. Davison, of the American Red Cross. The headquarters are at Geneva.—W. C. P.

### Government Hospitals in the East

FOR information as to the Italian Medical Service see the Educational Number of THE HOSPITAL, September 6, p. 578. By Government hospitals in the East we presume you mean British hospitals. You do not say whether you require a post as a nurse or in what capacity. You may obtain particulars from the High Commissioner in London of the particular Colony to which you desire to go. If you will write us further details of what you want to find we will do our best to help you.—H. G.

### Radiant Light and Heat.

THERE are many types of lamps for providing the treatment for conditions for which radiant light and heat are recommended, and most manufacturers of electro-medical apparatus can supply one or other of them. The Medical Supply Association, of Gray's Inn Road, make an excellent lamp for this purpose. The Stirling lamp manufactured by the Stirling Lamp Co., 39 South La Salle Street, Chicago, U.S.A., is a well-designed appliance, and is supplied in a series of sizes giving from 50 up to 2,000 c.p. The cost ranges from 9 to 42 dollars, exclusive of accessories.—W. H. F.

